# Influences of ventilation parameters on flow field and dust migration in an underground coal mine heading

**DOI:** 10.1038/s41598-020-65373-7

**Published:** 2020-05-22

**Authors:** Jianping Wei, Xiangyu Xu, Wan Jiang

**Affiliations:** 1 School of Safety Science and Engineering, Henan Polytechnic University, Jiaozuo, 454003 Henan China; 2 State Key Laboratory Cultivation Base for Gas Geology and Gas Control, Jiaozuo, 454003 Henan China; 3 Coal Production Safety Collaborative Innovation Center in Henan Province, Jiaozuo, 454003 Henan China

**Keywords:** Environmental sciences, Health occupations, Engineering

## Abstract

In order to effectively improve the efficiency of ventilation and dust removal, the flow field and dust migration characteristics under different ventilation parameters are deeply studied and analyzed by using the method of similar experiment and numerical simulation. Research results show that the change of the height of the ventilation duct affects the shape of the jet fluid, wind velocity distribution, the vortex distribution and the degree of disturbance of the airflow in the recirculation zone. When the height of the ventilation duct is *h* = 0.625 H (H is the height of the roadway), it shows a good ventilation and dust removal efficiency in roadway. The closer the ventilation duct is to the working face, the greater the eddy current intensity in the roadway and the more serious the dust accumulation. If the ventilation duct is 2 m away from the head, the dust concentration in roadway is relatively low. It shows a better ventilation and dedusting effect when the air velocity of ventilation duct *v* = 30 m/s, but it has a weak influence on the flow field structure in the roadway. The research results provide theoretical guidance for the selection, design and parameter optimization of the roadway ventilation and dust removal process.

## Introduction

According to the International Energy Agency (IEA)^1^, global coal power generation accounts for 37.93% of total power generation in 2018. So coal is still an important energy source. To date most of the coal production is from underground coal mining operation. In the case of underground mining, the working space is relatively confined. The dust generated after the coal machinery crushing is not easy to spread, especially the fine particles will be suspended in the tunnel air for a long time, then entering the alveoli through the human respiratory system and cause pneumoconiosis^2^. The diagnose of pneumoconiosis has accounted for more than 80% of occupational diseases in China in recent years^3^. Therefore, underground coal mine heading has become the most unsanitary working site in coal mining, and it has also become the focus of mine dust prevention.

In order to reduce the dust concentration in the roadway and ensure the health of the workers, coal seam water injection and spray dust reduction are common dust mitigation practices in underground coal mines. The coal seam water injection is to inject water into the coal seam through water injection drilling before mining and pre-wet the coal seam before mining. Meanwhile it can also help to reduce brittleness of coal body and proactively reduce production of coal dust during mining. The spray dust reduction is to install a spray device in the roadway or to equip the corresponding internal and external spray device in the shearer or roadheader^4^, using fine water droplets to capture and settle the dust suspended in the air. Although wet dust reduction can effectively reduce the dust concentration of roadway by 50~60%, sometimes even more than 90%^5^, there are still some problems in the process of wet dust removal: (1) water injection in coal seam is affected by the stress of the overlying bedrock and the influence of the porosity of the coal body and as a result, not all the coal seams are suitable for water injection; (2) dust reduction effect is affected greatly by the wettability of coal body, and the dust-reducing effect of coal with poor wettability is not good; (3) although too much water injection or excessive spray will have a good dust-reducing effect, it will weaken the strata and deteriorate the working environment; (4) wet dust reduction does not capture all the dust suspended in the roadway and itmust rely on a reasonable ventilation system to dilute and discharge the uncaptured dust to ensure the air quality in the working site.

In order to design a reasonable ventilation and dust mitigation system for tunnels, Candra *et al*.^6,7^ used Computational Fluid Dynamics (CFD) simulation to study the effects of different combinations of forced ventilation, exhaust ventilation and brattice on reducing the dust concentration of excavation work. Ren *et al*.^8^ proposed two feasible dust control schemes based on CFD numerical simulation to reduce the dust pollution above an underground bin. One is to modify the ventilation system to dilute the inhalable dust particles, another one is to use fine water mist filter suppresses and captures most of the dust particles. Geng *et al*.^9^ based on a simplified model of mixed ventilation simulated the diffusion law of dust particles with different particle sizes during coal roadway excavation, and analyzed the sedimentation, recirculation and dynamic distribution characteristics of dust. Cheng *et al*.^10^ used the combination of CFD-DEM coupling model and on-site measurement to study the flow field and the diffusion behavior of dust particles in force ventilation heading. Yu *et al*.^11^ adopted the same simulation method to study the diffusion pollution mechanism of high concentration dust in heading under force ventilation. Liu *et al*.^12^ and Mei *et al*.^13^ studied the distribution of dust in the roadway from the perspective of the flow field division of the roadway. Zhang *et al*.^14^ carried out experimental research on process technology of dust control and dust reduction on the high gassy tunneling face. In addition, many scholars apply similarity theory to the related engineering problems of particle and fluid coexistence^15,16^. Jiang *et al*.^17^ and Shi *et al*.^18^ used gas-solid two-phase flow theory to establish similarity criteria and studied the problem of ventilation and dust reduction on the working face and the heading face through similar experiments. At present, the related research focuses on the dust-reducing effect, which weakens the fundamental problem that the airflow is the main driving force of dust migration. The influence of the ventilation parameters on the flow field structure in the roadway has not been discussed in depth.

In this paper, both numerical simulation and similar experiments are employed to study the influence of ventilation parameters on the flow field and dust behavior. The influence of ventilation parameters on flow field are discussed and change of dust migration led by variation of ventilation parameters are deeply analyzed and studied. In order to obtain the reasonable ventilation and dust removal parameters in heading, the study provides theoretical and technical support for optimizing the ventilation and dust reduction system of the roadway.

## Similar simulation experiment platform construction and parameter setting

### Similarity theory

The airflow is the main driving force for the movement of dust particles. The combination of the gas motion equation and the dust particle motion equation is used to describe the physical phenomenon of gas-solid two-phase flow in the roadway^19^. The similar experiment is used to verify the migration process of the airflow and the dust in the real roadway, and the corresponding similarity criteria must be met.

The air in the tunnel is regarded as a viscous and incompressible fluid. For the unsteady flow of incompressible gas^15^, the equation of motion is given:

1 $${\rho }_{g}\left(\frac{\partial {V}_{g}}{\partial t}+{{\boldsymbol{V}}}_{{\boldsymbol{g}}}\nabla {{\boldsymbol{V}}}_{{\boldsymbol{g}}}\right)={\boldsymbol{m}}+{\boldsymbol{f}}-\nabla {\boldsymbol{P}}$$

Here ${\boldsymbol{m}}={\rho }_{g}{\boldsymbol{g}}$, ${\boldsymbol{f}}={\mu }_{g}{\nabla }^{2}{{\boldsymbol{V}}}_{{\boldsymbol{g}}}$, 且 ${\nabla }^{2}=\frac{{\partial }^{2}}{\partial {x}^{2}}+\frac{{\partial }^{2}}{\partial {y}^{2}}+\frac{{\partial }^{2}}{\partial {z}^{2}}$ Where: *ρ*_g_ is the gas density, kg/m^3^; ***V***_g_ is the gas velocity vector, m/s (*▽****V***_g_ is the velocity gradient); ***m*** is the mass force vector per unit volume, N/m^3^; ***P*** is the pressure vector of the gas, Pa (▽*P* represents pressure gradient); ***f*** is other force vector except mass force and gas pressure, N/m^3^; *g* is gravity acceleration vector, m/s^2^; *μ*_g_ is gas viscosity coefficient, N·s/m^2^.

The dust particles are considered to be spherical, and only the force generated by the relative motion of the gas-solid two phases is considered, and the equation of motion can be expressed as^15^

2 $$\frac{1}{6}\pi {d}_{s}^{3}{\rho }_{s}\frac{d{{\boldsymbol{V}}}_{s}}{dt}={C}_{D}\frac{1}{4}\pi {d}_{s}^{2}\frac{1}{2}{\rho }_{g}{{\boldsymbol{V}}}_{{\boldsymbol{r}}}|{{\boldsymbol{V}}}_{{\boldsymbol{r}}}|$$

Where: *d*_*s*_ is the diameter of the solid particles, m; *ρ*_*s*_ is the density of the solid particles, kg/m^3^; *C*_D_ is the drag coefficient; *V*_s_ is the velocity of the solid particles, m/s; *V*_r_ is the relative velocity of the gas-solid two phases, *V*_r_ = *V*_g_-*V*_s_.

**Figure 1:**
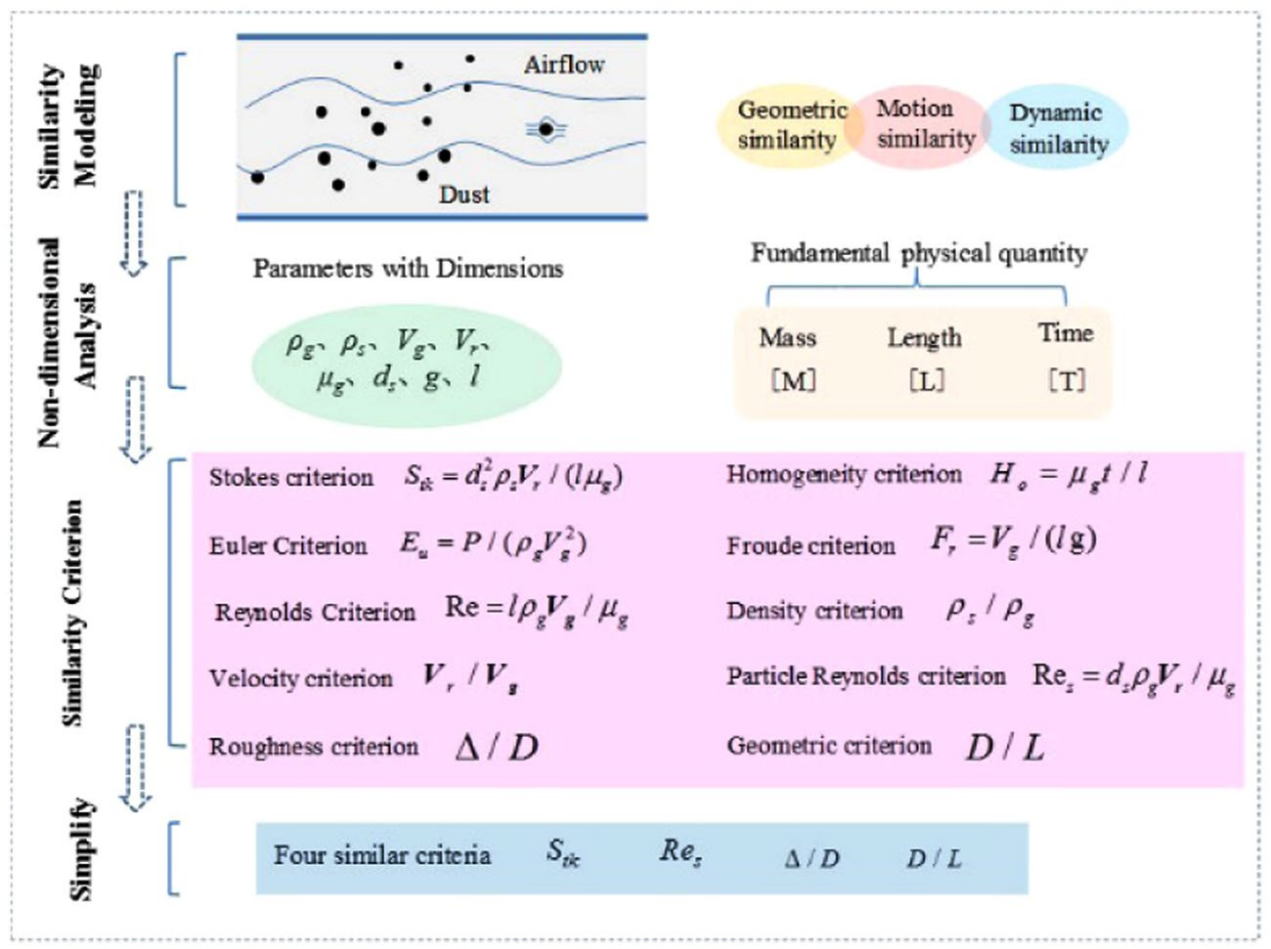
Schematic diagram of similarity theory of gas-solid two-phase flow.

In order to make the model experiment reflect the motion characteristics of the gas-solid two-phase in the roadway, the model experiment needs to satisfy the geometric similarity, dynamic similarity and motion similarity. Figure 1 shows the application of the similarity theory of gas-solid two-phase flow. The dimensionless analysis method is used to analyze the gas motion equation and the particle motion equation. The dimension quantities are ρ_g_, ρ_s_, V_g_, V_r_, μg, ds, g, l (characteristic length of geometry), t, P. The basic physical quantity is only mass [M], length [L] and time [T], then ten similarity criteria can be obtained according to the dimensional π theorem and the single-valued condition. In the actual research process, it is difficult to ensure that all similarity criteria are equal. Therefore, according to the need of ventilation and dust reduction in roadway, these ten similar criteria are simplified into four criteria: Stokes criterion *S*_tk_, particle Reynolds criterion *Re*_s_, roughness criteria and geometric similarity criteria^20^.

According to the first theorem of similarity theory, the two similar phenomena must have the same number of criteria for the same name^21^. $\Delta /D$ and $D/L$ is geometric similarity criteria, easy to achieve equal. The model is equal to the *S*_tk_ and *Re*_s_ of the prototype. The simultaneous equations can be used to derive the relationship between the wind velocity *V*_gm_ in the model and the prototype wind velocity *V*_gy_ (subscript y for prototype and subscript m for model).

3 $${{\boldsymbol{V}}}_{{\boldsymbol{gm}}}=\frac{{l}_{y}}{{l}_{m}}{\left(\frac{{\rho }_{gy}}{{\rho }_{gm}}\right)}^{2}\frac{{\mu }_{gm}}{{\mu }_{gy}}{{\boldsymbol{V}}}_{{\boldsymbol{gy}}}$$

Known, $$\mathrm{Re}=\frac{l{\rho }_{g}{{\boldsymbol{V}}}_{{\boldsymbol{g}}}}{{\mu }_{g}}$$, then

4 $$\frac{{\mathrm{Re}}_{m}}{{\mathrm{Re}}_{y}}=\frac{{l}_{{\rm{m}}}}{{l}_{y}}\frac{{\rho }_{gm}}{{\rho }_{gy}}\frac{{\mu }_{gy}}{{\mu }_{gm}}\frac{{{\boldsymbol{V}}}_{gy}}{{{\boldsymbol{V}}}_{gm}}$$

Take Eq. (3) into Eq. (4), then $\frac{{\mathrm{Re}}_{m}}{{\mathrm{Re}}_{y}}=\frac{{\rho }_{gy}}{{\rho }_{gm}}$

From the above equation, the ratio of the Reynolds number of the model to the prototype is equal to the inverse ratio of the airflow density. The density difference between the mine air and the ground air is small, so it can be realized, the model has essentially the same fluid Reynolds number as the prototype, under the conditions of satisfying the Stokes criterion and the particle Reynolds number criterion. Meanwhile the fluid Reynolds number Re > 2300 should be satisfied, and the fluid is in a completely turbulent state. Both the prototype and the model fluid are located in the second self-modulation zone, ensuring similar dynamics and similar fluid motion.

### Construction and parameter setting of similar simulation platform

**Figure 2:**
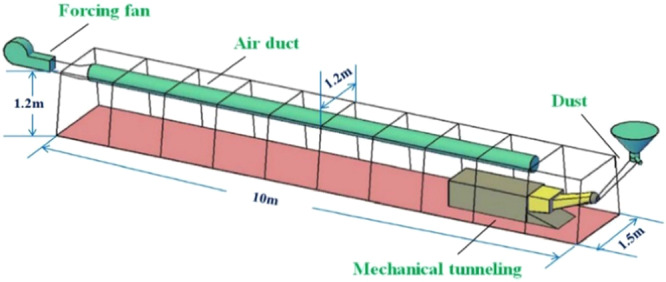
A plan of the similar model of driving roadway.

**Figure 3:**
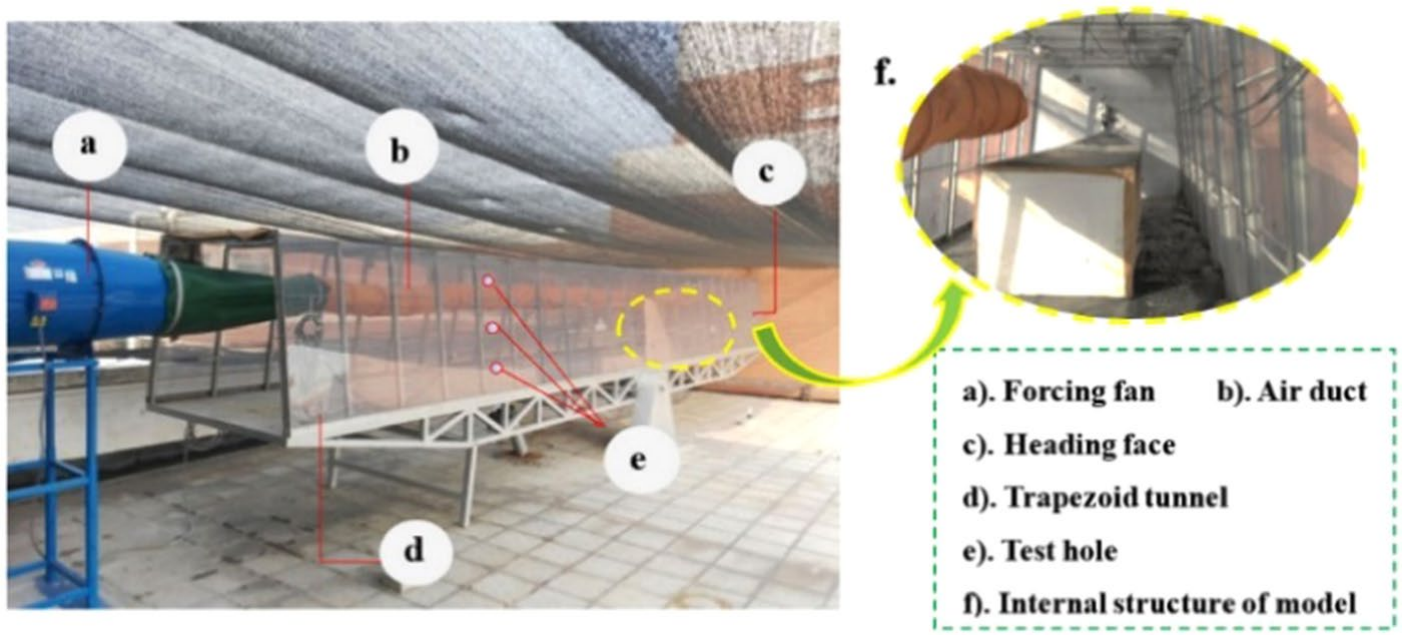
The platform similar experimental system.

**Figure 4:**
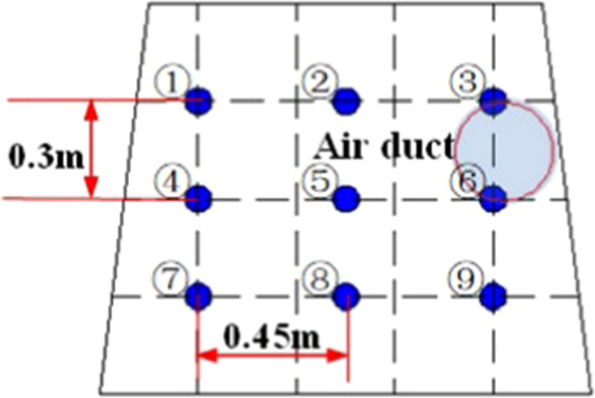
The distribution of test points of air velocity.

**Figure 5:**
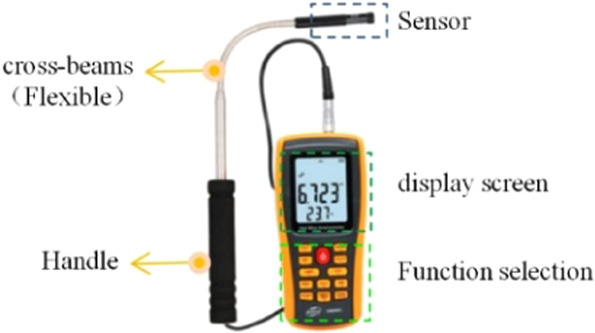
The thermal anemometer.

Based on the above theory, the authors designed and built a similar model experimental platform for the roadway, as shown in Figs. 2 and 3. The similar model is an isosceles trapezoidal roadway with a length of 10 m. The walls on both sides of the roadway model are made of transparent plexiglass material, which is easy to observe, and the rest are steel frame structure. The trapezoidal section size is 1.2 m for the upper base, 1.5 m for the lower base, and 1.2 m higher. The diameter of the ventilation duct is 0.3 m, and the model of the roadheader is simplified by the EBZ120 type roadheader. The size is 2.7 m long, 0.6 m wide and 0.5 m high. The selection of the model dimensions is based on the consideration of the real size of the common trapezoidal roadway and the limited test site space, and finally it is determined by combining the appropriate geometric similarity ratio. There are nine wind velocity measuring points in the same section of the roadway. The wind velocity measuring point distribution is shown in Fig. 4. The wind velocity measuring instrument is Biaozhi thermal anemometer GM8903. As shown in Fig. 5, the instrument accuracy is 0.001 m/s, which is connected with the sensor. The probe can be stretched and bent. During the experiment, the probe is passed through the test hole of the wall of the roadway, and the wind velocity at different positions is determined by adjusting the length and the angle.

Based on the above geometry, the relevant parameters can be determined as follows: It is considered that the air density in the roadway is equal to the model experimental gas density, and the air density at 20 °C is taken, that is, *ρ*_gy_ = *ρ*_gm_ = 1.205 kg/m^3^; the prototype gas dynamic viscosity coefficient of the roadway is *μ*_gy_ = 2.3 × 10^−5^N·s/m^2^, model gas dynamic viscosity coefficient *μ*_gm_ = 1.81 × 10^−5^N·s/m^2^; geometric size ratio (the ratio between the model size and the corresponding size of the prototype.) is 0.3, model equivalent diameter *D*_m_ = 1.266 m. The actual average wind velocity of the roadway is generally 0.25~1 m/s. According to Eq. (3), the average wind velocity range of the similar experiment of the force-in ventilation of the roadway can be calculated.

From the above conditions, we can know:

$${l}_{m}/{l}_{y}=0.3,\,{\rho }_{gm}/{\rho }_{gy}=1,\,{\mu }_{gm}/{\mu }_{gy}=0.78,\,{\rho }_{sm}/{\rho }_{sy}=1,$$

$${{\boldsymbol{V}}}_{{\boldsymbol{gm}}}=\frac{10}{3}\times {1}^{2}\times 0.78{{\boldsymbol{V}}}_{{\boldsymbol{gy}}}=2.62\times (0.25 \sim 1)\,{\rm{m}}/{\rm{s}}$$

and,

$${\mathrm{Re}}_{y}={\mathrm{Re}}_{m}=\frac{{D}_{m}{\rho }_{gm}{{\boldsymbol{V}}}_{{\boldsymbol{gm}}}}{{\mu }_{gm}}=1.74\times {10}^{5}$$

Re > 2300, The similar experimental conditions were met, so the average wind velocity of the model experiment was controlled in the range of 0.66~2.62 m/s.

## Numerical model

### Numerical model and boundary conditions

In this paper, the Euler-Lagrangian model is used to calculate the flow field and dust migration law in the roadway^22^. The Euler-Lagrangian method treats the fluid as a continuous phase and applies Newton’s second theorem to track the solution flow^23^. The basic governing equations for particle motion trajectories, flow fields and dust migration in the field are as follows^24^: (1) The gas phase continuity equation in gas-solid two-phase flow is

5 $$\frac{\partial }{\partial t}{\rho }_{g}+\frac{\partial }{\partial {x}_{i}}({\rho }_{g}{u}_{j})=0$$

Where:* t* represents time; *ρ*_g_ is gas density; u represents time average velocity; i, j represents the direction in the free coordinate system.

(2) The momentum conservation equation is

6 $$\frac{\partial }{\partial t}({\rho }_{g}{u}_{i})+\frac{\partial }{\partial {x}_{i}}({\rho }_{g}{u}_{i}{u}_{j})=\frac{\partial }{\partial {x}_{j}}({\alpha }_{f}{\tau }_{ij})-\frac{\partial p}{\partial {x}_{i}}+{F}_{sf}+{\rho }_{g}g$$

Where: *p* is the normal stress; tangential stress; *F*_sf_ is the force of the discrete particles on the fluid.

(3) The Realize k-ε model equation in gas-solid two-phase flow mode is:

7 $$\frac{\partial }{\partial t}(\rho k)+\frac{\partial }{\partial {x}_{j}}(\rho k{u}_{j})=\frac{\partial }{\partial {x}_{j}}\left[\left(\mu +\frac{{\mu }_{\tau }}{{\sigma }_{k}}\right)\frac{\partial k}{\partial {x}_{j}}\right]+{G}_{k}+{G}_{b}-\rho \varepsilon -{Y}_{M}+{S}_{k}$$

8 $$\frac{\partial }{\partial t}(\rho \varepsilon )+\frac{\partial }{\partial {x}_{j}}(\rho \varepsilon {u}_{j})=\frac{\partial }{\partial {x}_{j}}\left[\left(\mu +\frac{{\mu }_{\tau }}{{\sigma }_{\varepsilon }}\right)\frac{\partial \varepsilon }{\partial {x}_{j}}\right]+\rho {C}_{1}S\varepsilon -\rho {C}_{2}\frac{{\varepsilon }^{2}}{k+\sqrt{v\varepsilon }}+{C}_{1\varepsilon }\frac{\varepsilon }{k}{C}_{3\varepsilon }{G}_{b}+{S}_{\varepsilon }$$

Where: *k* represents enthalpy flow energy; ε represents dissipation rate; *μ* is viscosity; *μ*_t_ is turbulent viscosity; G_b_ is turbulent flow energy due to buoyancy; *G*_k_ is the generation phase of turbulent energy k due to average velocity gradient; *Y*_M_ is caused by transporting the fluctuation of the compressible turbulence generated by shifting and diffusion; ${\sigma }_{k}$ is the Planton number corresponding to the turbulent energy *k*; *S*_k_ is the generation term of the turbulent energy k caused by the movement of the particles; ${S}_{\varepsilon }$ is the generation rate of the dissipation rate due to the particle phase; C_1_, C_2_, and C_3_ are empirical constants.

(4) According to Newton’s second law, the equation for the motion of the particle term is:

9 $${m}_{p}\frac{{\rm{d}}{v}_{p}}{{\rm{d}}t}={F}_{fp}$$

10 $${I}_{p}\frac{{\rm{d}}{w}_{p}}{{\rm{d}}t}={M}_{fp}$$

Where: *m*_p_ is the mass of the particle, *v*_p_ is particle velocity; *F*_fp_ is the fluid force of the continuous gas phase and the particle; *I*_p_ is the inertial term of the particle; wp is the angular velocity of the particle rotation; M_fp_ is the total moment of rotation acting on particles.

**Figure 6:**
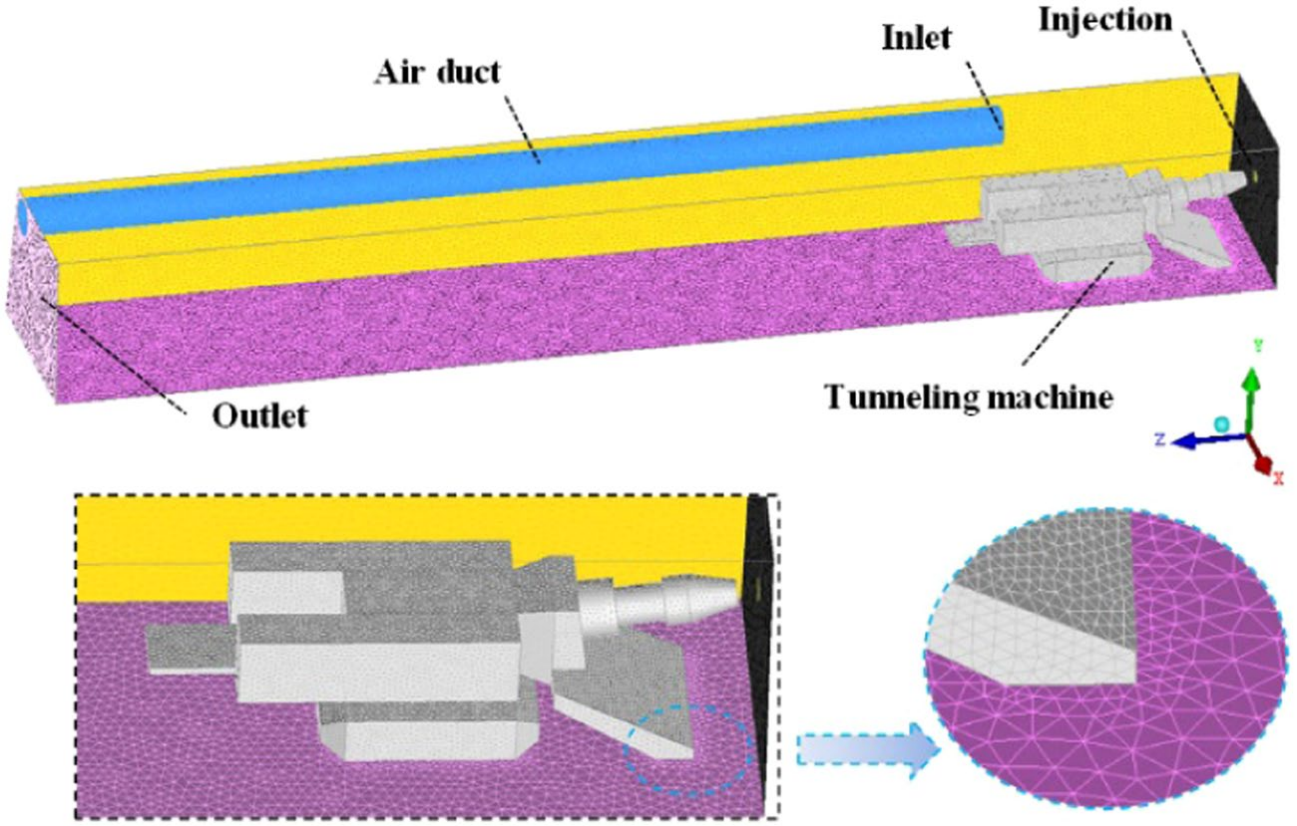
Physical model and grid of roadway.

ICEM CFD18.0 (The Integrated Computer Engineering and Manufacturing code for Computational Fluid Dynamics) is used to draw a physical model of the same size as the similar experimental platform, the model is shown in Fig. 6. Compared with the experimental system, the fan is simplified, and only the ventilation duct is reserved as the air source; and reduce the calculation load, the flow field inside the ventilation duct is not within the calculation domain; the model X axis represents the roadway width, and the Y axis represents the roadway height. The Z axis represents the length of the roadway. The computational grid is mainly composed of unstructured cells. Because the body of the roadheader has more corners, the corresponding mesh is encrypted in the vicinity of the roadheader, and the grid is also encrypted near the wall of the ventilation duct and the outlet of the ventilation duct. The total number of grids is around 1145,000, and the average grid quality is 0.745, which meets the calculation accuracy requirements. The minimum mesh quality is 0.3 and the maximum mesh quality is 0.9995.

The simulation method settings are shown in Table 1. The k-ε equation is used to simulate the fluid phase motion of the press-in ventilation, and the dust is regarded as a discrete phase, which is solved by the DPM model and coupled with the continuous phase. The particle tracking method is non-stationary particle tracking, which can obtain the particle distribution at a certain moment. The calculation model assumes the energy equation without considering the heat exchange problem.

**Table 1:** Simulation method settings.

**General**	
Solver	type: pressure-based; velocity formulation: absolute; time: steady
**Models**	
Energy	off
Viscous model	model: k-epsilon(2eqn); k-epsilon model: Realizable; Near-wall treatment: Standard wall functions
Heat exchanger	off
Discrete Phase model	Interaction: interaction with continuous phase; particle treatment: unsteady particle tracking; Stochastic tracking: discrete random walk model
**Solution**	
pressure-velocity coupling	SIMPLE

The boundary mainly includes the inlet, the outlet, the particle jet source, and the wall surface. The inlet of the ventilation duct is the source of wind flow. The set conditions include the inlet wind velocity and the turbulence intensity. For the inlet wind velocity of 30 m/s, the corresponding turbulence intensity is 3.03%. Since the exterior of the similar experimental roadway is an atmospheric environment, the outlet can be set to be outflow. The dust source is located on the roadway surface corresponding to the head of the roadheader, and the corresponding mass flow rate and particle properties are set. The specific parameters are shown in Table 2.

**Table 2:** Boundary conditions.

**Inlet**	
Type	velocity inlet
Specification method	Intensity and Hydraulic Diameter
**Outlet**	
Type	outflow
**Wall**	
Shear condition	no slip
DPM-boundary type	reflect/ escape
**Injection properties**	
Injection type	surface (round); area:0.00785m^3^
Material	coal-hv; particle density:1400 kg/m^3^
Total flow rate	0.0012 kg/s
Diameter distribution	type: rosin-rammler; min. diameter:1μm; max. diameter:100μm; mean diameter:0.48μm; spread parameter: 3.5; number of diameters:15

### Base model result

**Figure 7:**
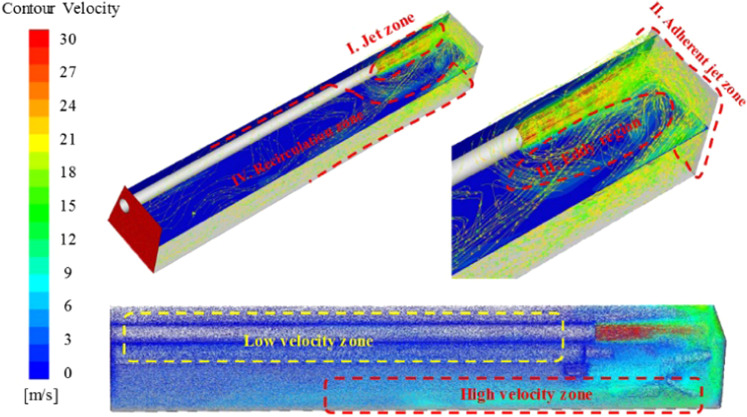
Roadway flow field map.

**Figure 8:**
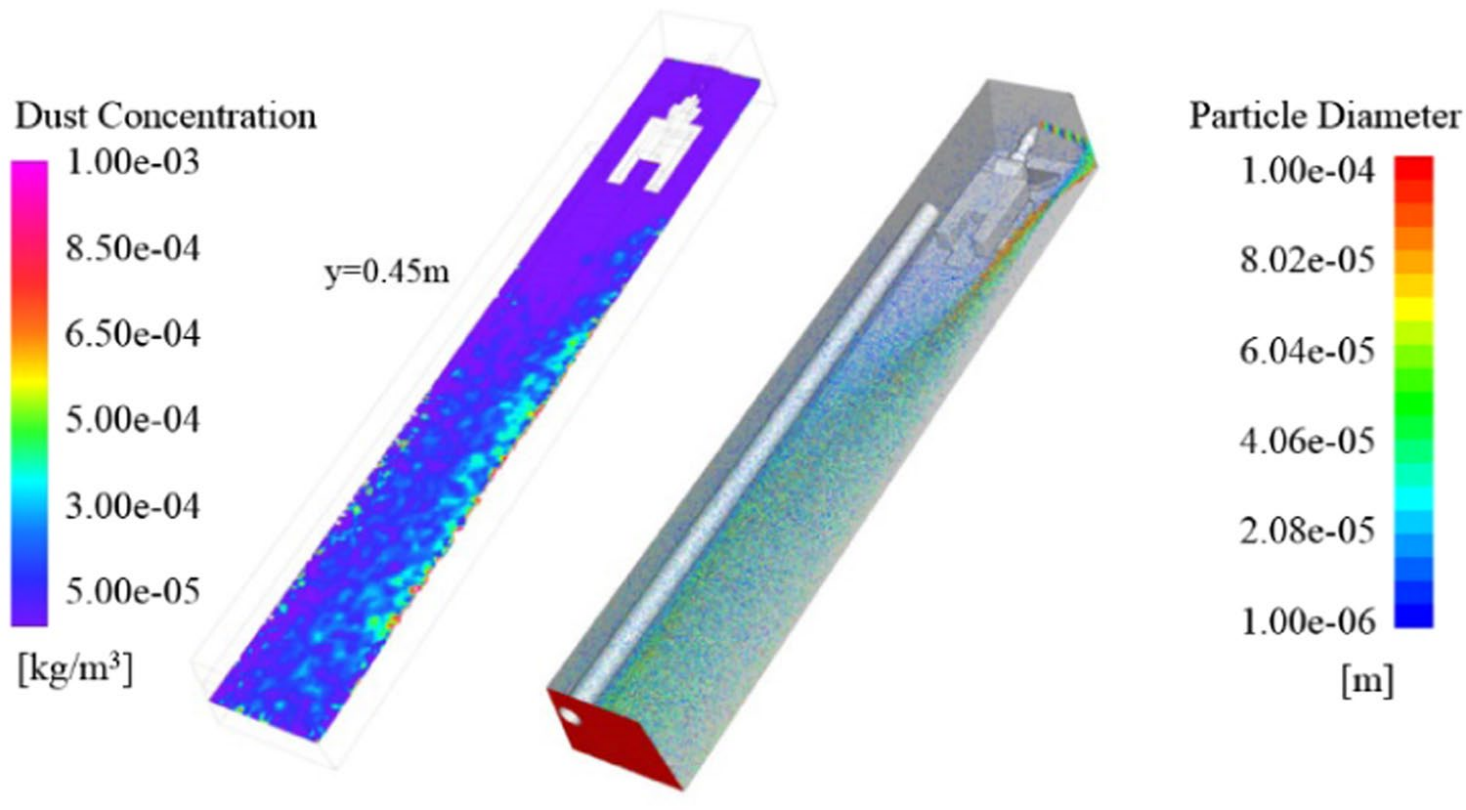
Dust simulation results of basic model.

The basic ventilation parameters of this model are as follows: the ventilation duct is 0.75 m from the bottom of the roadway, 2 m from the head, 0.07 m from the wall, and the air velocity of the ventilation duct is 30 m/s. The flow field characteristics and dust migration of the tunneling are calculated through Fluent 18.0 solver. After the fresh air is ejected from the ventilation duct, it hits head-on and then turns back. Most of the airflow moves along the roof and wall of the roadway and flows to the rear of the roadway. According to the development process of the wind flow in the roadway, it is divided into four part: Jet zone, adherent zone, eddy region and recirculation zone, as shown in Fig. 7. The simulation results of dust distribution are shown in Fig. 8, including the dust concentration distribution and roadway dust particle size distribution in the section of 0.45 m.

Area I in Fig. 7 is located in front of the air outlet of the ventilation duct, where the air velocity is high but attenuates rapidly. The jet zone is mainly based on the development of the jet, with a more regular flow field structure. The adherent jet zoon of area II in Fig. 7 is located near the head of the roadway. The airflow from the jet zone forms an attached flow on the wall and then turns back. In the attached jet zoon, the airflow velocity attenuates greatly and has variable airflow directions. The eddy region of area III in Fig. 7 is mainly located near the fully mechanized excavator, where the structure of the flow field is complex. There are many vortex structures, which is caused by the entrainment effect of the jet zone and the space confined by the roadway wall and the roadheader. The recirculation zoon is located on the right side and rear of the roadheader, as shown in area IV in Fig. 7. The flow field structure is relatively simple, and the airflow flows to the exit of the roadway. The characteristic is that the air velocity near the wall of return side is higher than that of the middle roadway and the side of the ventilation duct.

As can be seen from the Fig. 8, the area with higher dust concentration in the section of breathing height is in the recirculation zoon. The dust concentration near the fully mechanized excavator is low. In this case, the visibility of the working face is relatively high, which is conducive to the safe operation of coal workers. Combined with the particle size distribution, the dust content in the jet zone is the lowest, because there is a large amount of fresh air entering here. The air velocity in the recirculation zone on the right side of the fully mechanized excavator is high, which can carry large particles of dust. Most of the dust trapped in the eddy region are fine particles.

### Model validation

**Figure 9:**
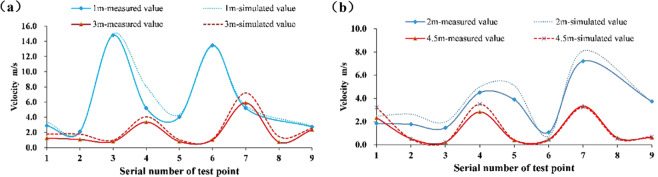
Comparison of measured and simulated wind velocity in different sections. (**a**) Comparison of measured wind velocity and simulated wind velocity in 1m and 3m sections. (**b**) Comparison of measured wind velocity and simulated wind velocity in 2m and 4.5m sections.

The numerical simulation results were verified by experiments using similar platforms. Since the air velocity of the roadway has a certain fluctuation, each measuring point in the experiment is measured three times and take the average value. The wind velocity of each measuring point in the four cross-sections of the headway 1 m, 2 m, 3 m and 4.5 m is measured. The comparison between the simulation results and the experimental results is shown in Fig. 9. The numerical simulation wind velocity of the same section in the roadway is basically the same as the experimental result. The wind velocity values of the very few measuring points are different, mainly due to the experimental conditions and errors, and the overall data fit is better.

**Figure 10:**
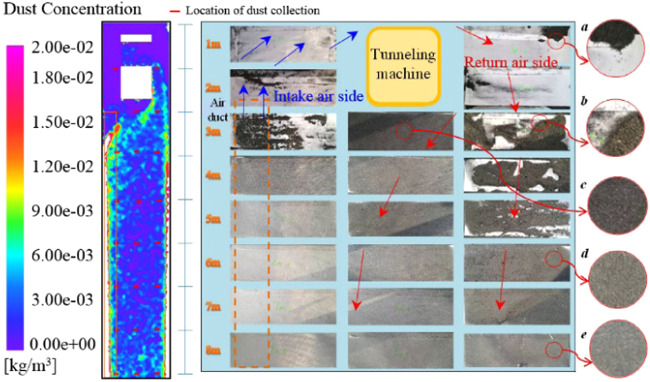
Simulation and experimental results of dust distribution on floor.

Except for the 1 m section which is greatly affected by the jet zoon, the air velocity of 1, 4 and 7 measuring points in the same section of the roadway are all higher than those of other measuring points. That is, the air velocity on the return side is larger than that in the middle of the roadway and the ventilation duct side, which is consistent with the numerical simulation results. After passing through the dust source, the airflow carries the dust around the fully mechanized excavator to the rear of the roadway. It can be considered that the settling characteristics of floor dust are related to the distribution of wind speed. Therefore, the law of dust settlement is observed by evenly placing white paper on the roadway floor. The experimental results and simulation results are shown in Fig. 10. The chart shows that most of the dust settles on the return side, and there are large particles of dust at the distance of 3 m to 5 m. The particle size of dust decreases gradually from a to e. In addition, the dust carried by the airflow bypassing the fully mechanized excavator also settles. The dust deposition characteristics of the above experiments are basically consistent with the dust concentration distribution of the simulation results. Because of the high wind speed on the return side, it can carry most of the large particles to migrate and settle on the return side. The dust with smaller particles has less inertia and spreads to the bottom of the wind tube with the disturbed airflow. In addition, the simulation results show that the dust concentration is the highest on both sides of the floor, which is due to the high resistance at the corner of the roadway.

### Parametric study

The ventilation parameter is the influencing factor that causes the change of the flow field structure of the roadway. The change of the height of the ventilation duct, the position of the ventilation duct and the wind velocity will cause the change of the flow field structure in the roadway, thus affecting the dust migration. To this end, relevant parameters research was carried out: (1) studying the influence law of the height of the ventilation duct on the flow field structure and dust movement of the roadway; (2) studying the influence of the position of the ventilation duct on the flow field structure and dust movement of the roadway; (3) Study the influence of different wind velocity on the dust reduction efficiency of roadway.

### Height of duct

The ventilation duct is set to the head *l* = 2 m, the wind velocity at the air outlet of the ventilation duct is 30 m/s, and the numerical simulation of the flow field structure of the tunnel under the conditions of *h* = 105 cm, 75 cm, 45 cm and 15 cm respectively.

**Figure 11:**
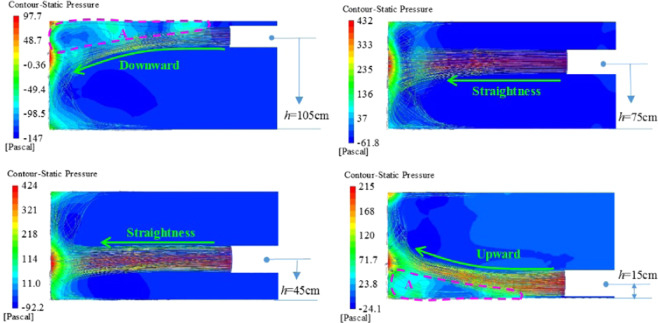
Wind flow trace and pressure cloud diagram of the central section of the air duct at different heights.

Figure 11 is a wind flow trace and pressure cloud diagram of the center section of the ventilation duct of different ventilation duct heights. In the jet flow area, when the ventilation duct is at a high position, the main body jet is downwardly shifted; when the height is at a low position, the main body jet is upwardly offset. When the ventilation duct is located in the middle of the roadway, the main body of the jet does not substantially shift. The main reason is that the area A in the figure is a confined space composed of the roof, the wall and the working surface of the roadway. The high-velocity airflow passes through the A area, which will impact and compress the still air in the area, making it a high-pressure area, hindering The airflow moves toward the A region, forcing the jet body to shift downward. Similarly, the jet body is offset upward when the ventilation duct is at the bottom. When the ventilation duct is located in the middle of the roadway, the influence of the confined space on its airflow movement is relatively small, so that the main body of the jet does not substantially shift.

At the same time, when the ventilation duct is located at the top or bottom of the roadway, the high pressure gas in A area of the roadway will hinder the development of the jet. In order to overcome the anti-impact effect of the high pressure gas from the A area, the fluid kinetic energy loss in the jet area will be caused. At the same time, the fluid in the opposite direction around the jet zone will enter the jet body due to space limitation and the entrainment of high-velocity gas, further increasing the loss of fluid kinetic energy in the jet region; while the ventilation duct is located in the middle of the tunnel, the gas flow energy loss will be greatly reduced, because the high-pressure gas formed by the jet section in the headway impact will spread to the surrounding, and the anti-impact effect on the jet gas is small. At the same time, when the ventilation duct is located in the middle of the roadway, the space is relatively open, the space limitation is not obvious, the reverse impact fluid does not enter the main body fluid, the entrainment effect of the high velocity gas only brings the still air around the jet into the jet body, which is relatively small for the kinetic energy loss of the fluid in the jet zone. Therefore, in order to reduce the attenuation of the dust exhaust wind velocity in the roadway, the ventilation duct should not be too high or too low.

**Figure 12:**
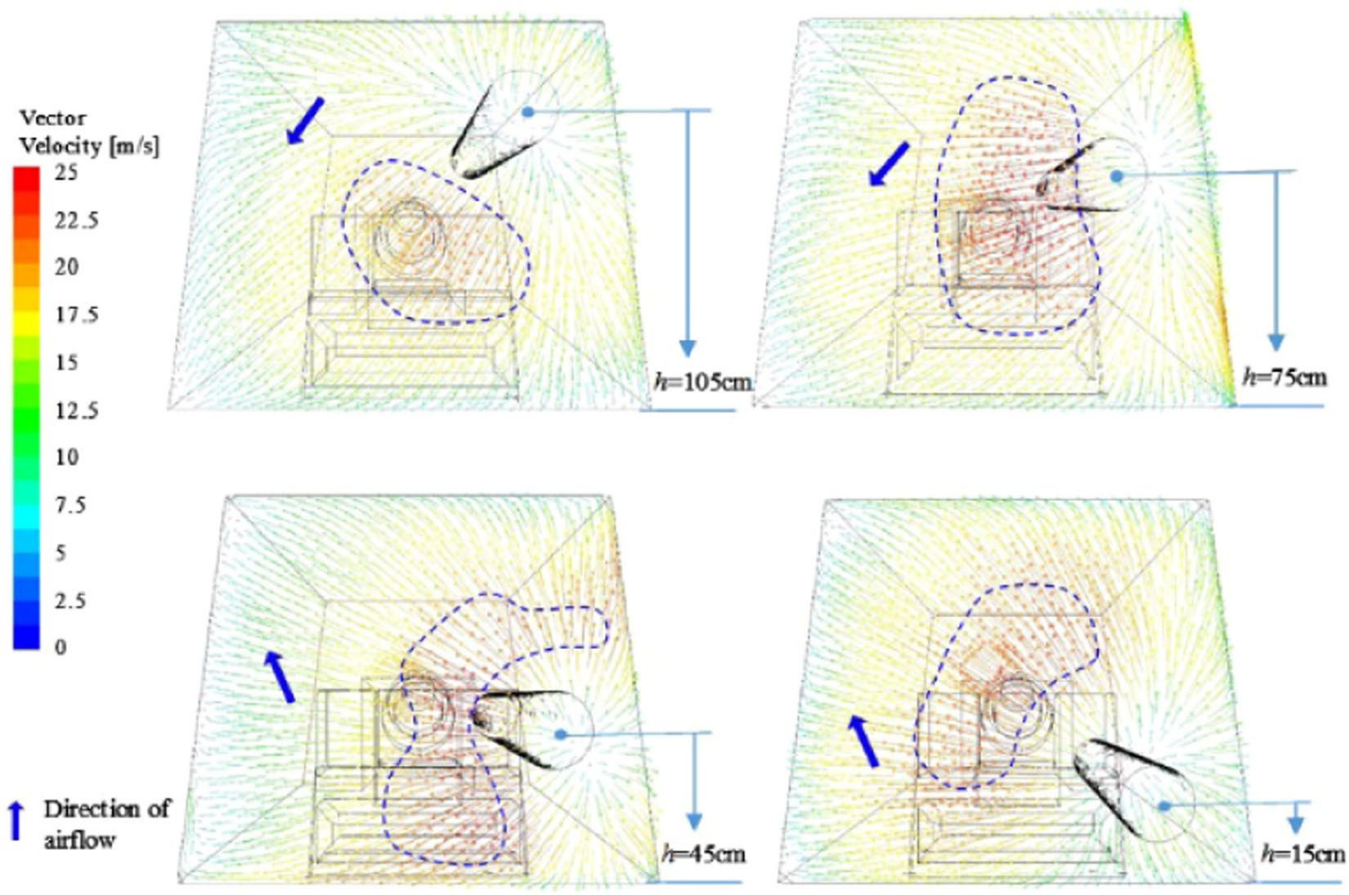
Roadway attachment area wind velocity vector of different ventilation duct height.

Figure 12 is a wind flow trace diagram of the roadway attachment area of different ventilation duct heights. The dotted line range is the area where the wind velocity is higher in the attached jet zone. When the ventilation duct is at the highest and lowest, the high-velocity wind flow area is relatively small, and when the ventilation duct located in the middle, the high-velocity wind flow area is large, and the wind velocity is instantaneously slow. The main reason is due to the attenuation of wind flow energy in the jet area. With the change of the height of the ventilation duct, the direction of the wind flow also changes accordingly. When the ventilation duct is located at the height of the roadway, the main body of the wind flow moves toward the bottom of the roadway. When the ventilation duct is at the bottom of the roadway, the main body of the wind flow moves toward the top of the roadway. The dust in the roadway is mainly from the attachment area. The airflow direction determines the diffusion range of the dust in the roadway. When the airflow direction is opposite to the sedimentation direction of the dust, the dust is extremely easy to spread in the roadway and is not easy to settle.

**Figure 13:**
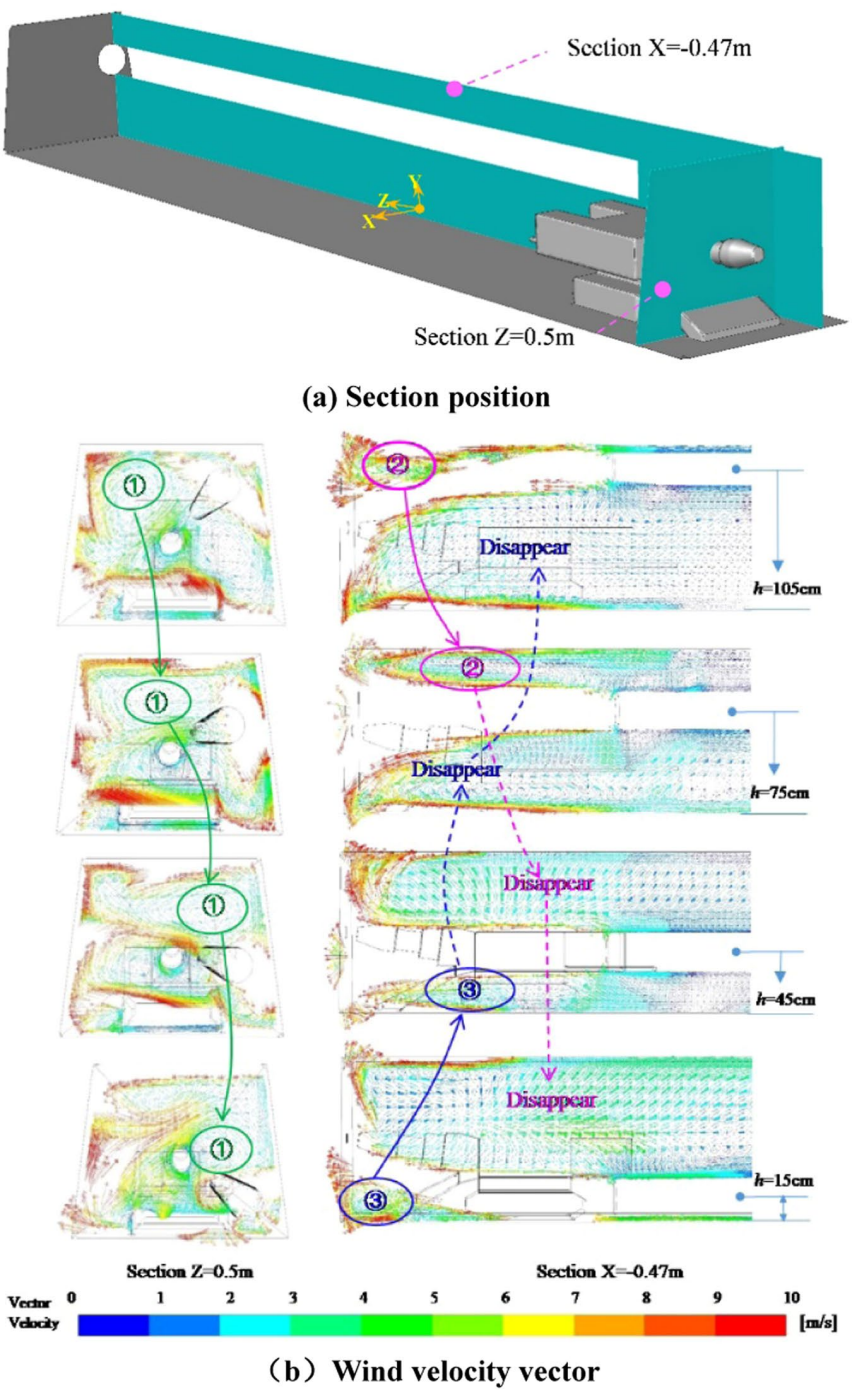
Distribution of eddy current position in vortex area in the roadway of different duct heights.

The flow field structure in the eddy current zone is disordered, which is the main area for the formation of eddy currents, and the formation of eddy currents is caused by the entrainment action and space limitation of the high-velocity airflow. Figure 13 is a wind velocity vector diagram of the roadway with X = −0.47 m and Z = 0.5 m sections at different duct heights. The distribution of the vortex is marked in the figure. It can be found that the height of the duct is related to the position distribution of the vortex. The formation of the vortex No.1 is caused by the entrainment action of the high-velocity fluid, and the position of the vortex is gradually shifted downward as the height of the ventilation duct decreases; The No.2 vortex is formed by the restricted space in the upper part of the tunnel when the ventilation duct is at the highest position. As the height of the ventilation duct decreases, the spatial confinement becomes weaker, which is formed by the interaction of the spatial confinement and the entrainment of high-velocity fluid. When the height of the ventilation duct continues to decrease, the space-constrained effect is gradually lost, and the high-velocity airflow is insufficiently sucked to form a vortex, which eventually leads to the disappearance of the No. 2 eddy current. The No. 3 eddy current is opposite to that. As the downward movement of the ventilation duct is gradually enhanced by the limited space in the lower part of the roadway, the vortex of the No. 3 vortex gradually forms. Therefore, the main eddy current position in the vortex zone is synchronized with the height of the roadway, that is, when the ventilation duct is at a high position, the main eddy current is also at a high position, and vice versa.

**Figure 14:**
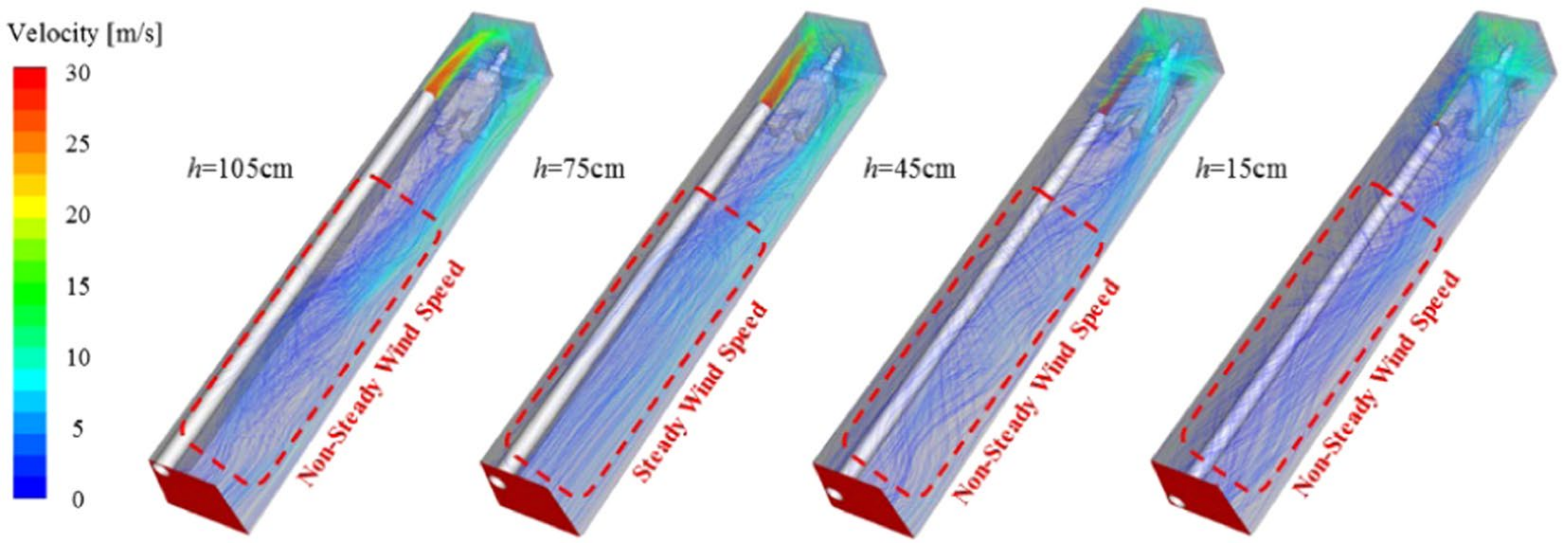
Roadway wind flow map under different ventilation duct height conditions.

After the airflow from the ventilation duct passes through the jet zone, the attached jet zone and the vortex zone, the wind flow body gradually diffuses toward the rear of the roadway to form a recirculation zone. The wind flow trace of the roadway under different ventilation duct height conditions is shown in Fig. 14. When the ventilation duct is 75 cm away from the bottom plate, the wind flow traces in the recirculation zone of the roadway are mostly parallel to the roadway, and the flow field structure is stable. However, in the other three working conditions, the wind flow field structure is relatively disordered. The reason is analyzed: when the ventilation duct is at the top of the roadway or at a relatively low position, the main body of the wind flow is relatively close to the wall surface of the roadway, and the airflow and the wall surface will impact and change its direction, so that the flow field structure in the recirculation zone becomes disordered. At the same time, friction will occur with the wall surface of the roadway to increase the kinetic energy loss of the wind flow and reduce the efficiency of ventilation and dust exhaust in the roadway. Therefore, when performing roadway ventilation and dust removal, the ventilation duct should not be too high or too low.

**Figure 15:**
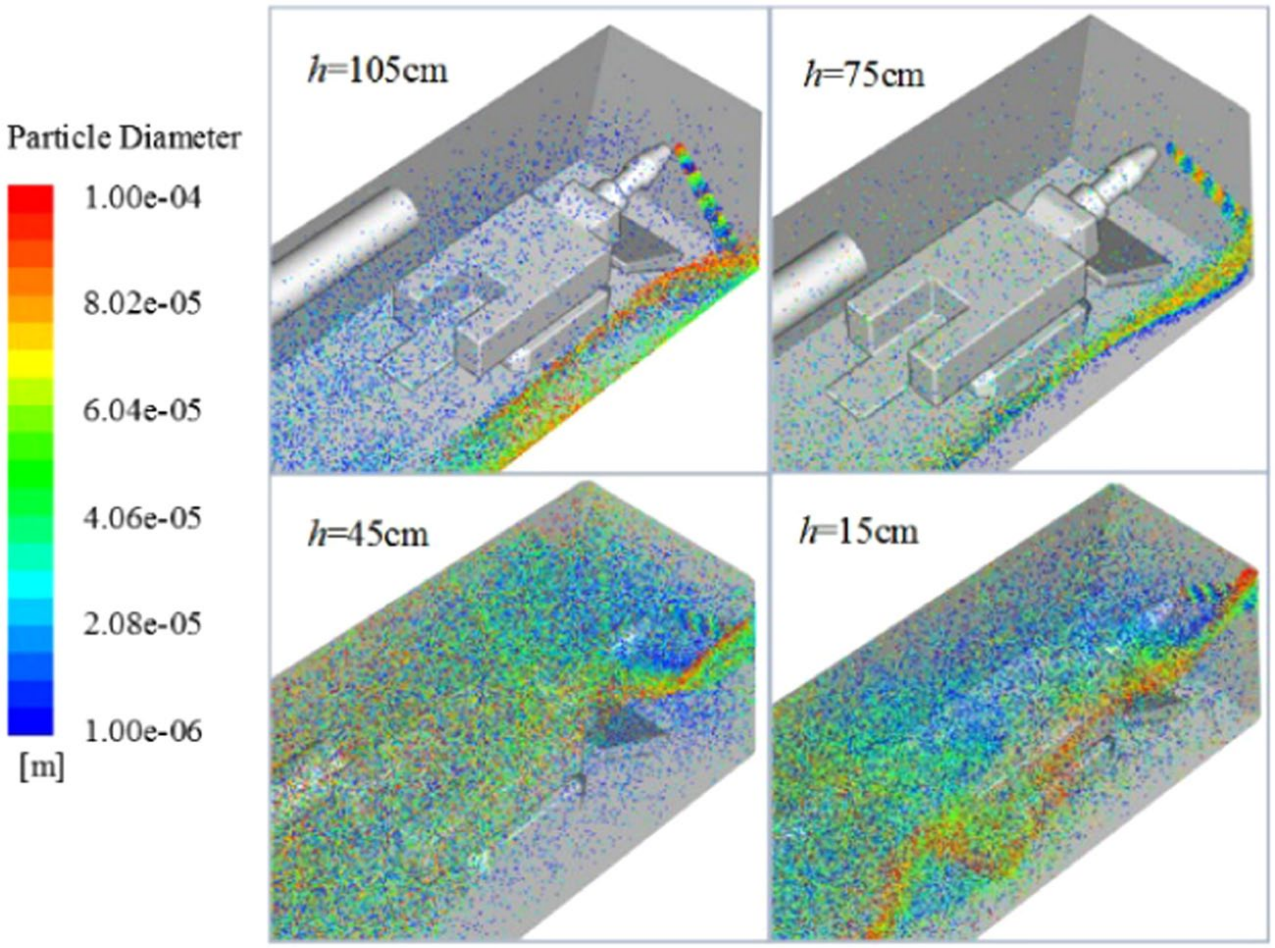
Flow field and dust particle distribution of the heading face under different ventilation duct heights.

Regardless of the height of the ventilation duct, the flow field structure in front of the roadway is disordered, and the main flow direction of the air flow is different. Figure 15 shows the dust particle distribution map of the roadway under different ventilation duct heights. Generally speaking, when the ventilation duct is located in the middle and upper part of the roadway, the air quality in the roadway is better than the ventilation duct in the lower part of the roadway. The main reasons are as follows: when the ventilation duct is at the middle and upper part, the wind flow flows from the upper part of the roadway to the lower part of the roadway, which is consistent with the direction of sedimentation of the dust, and when the ventilation duct is located at the lower part, the wind flow flows upward from the lower part of the roadway, contrary to the direction of sedimentation of dust, it is easy to form dust; secondly, combined with the structural characteristics of the flow field in front of the roadway, the lower the position of the ventilation duct, the more complicated the flow field structure in front of the roadway, the more impacts on the wall surface of the wind flow and the roadway, and the wind velocity decays faster, dust stays in space for a long time.

In order to compare the dust removal effects of different ventilation duct heights, the proportion of escape dust at the exit of the roadway to the total amount of dust is counted. As shown in Table 3, from the perspective of the escape ratio of dust at the exit of the roadway, the exhaust efficiency is best when the ventilation duct is 75 cm away from the bottom plate. At the same time, by comprehensively analyzing the wind flow field structure, wind velocity attenuation, dust diffusion and ventilation dust removal efficiency in the roadway, the height of the ventilation duct in the roadway should not be too low, and when the center of the ventilation duct is located at 0.625 H (H is the roadway height), the roadway ventilation and dust reduction efficiency is best.

**Table 3:** The proportion of escaped dust.

Working condition	Air cylinder to the bottom plate 105 cm	Air cylinder to the bottom plate 75 cm	Air cylinder to the bottom plate 45 cm	Air cylinder to the bottom plate 15 cm
Number Tracked	64	64	62	64
Number Escaped	32	34	26	23
Proportion/%	50	53	42	36

### Location of duct outlet

Set the height of the ventilation duct *h* = 75 cm and the exit velocity of the ventilation duct to 30 m/s. According to the working experience, generally, the distance from the duct to the heading is less than (4~5), and S is the cross-sectional area of the roadway. Therefore, the numerical simulation is carried out to study the variation of flow field and dust migration in the roadway under the conditions of the distance *l* = 1 m, 2 m, 3 m, 4 m.

**Figure 16:**
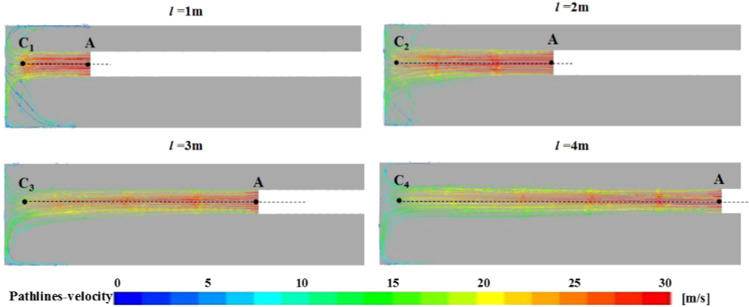
Wind flow trace of the section of the tunnel in the jet zone at different ventilation duct positions.

**Figure 17:**
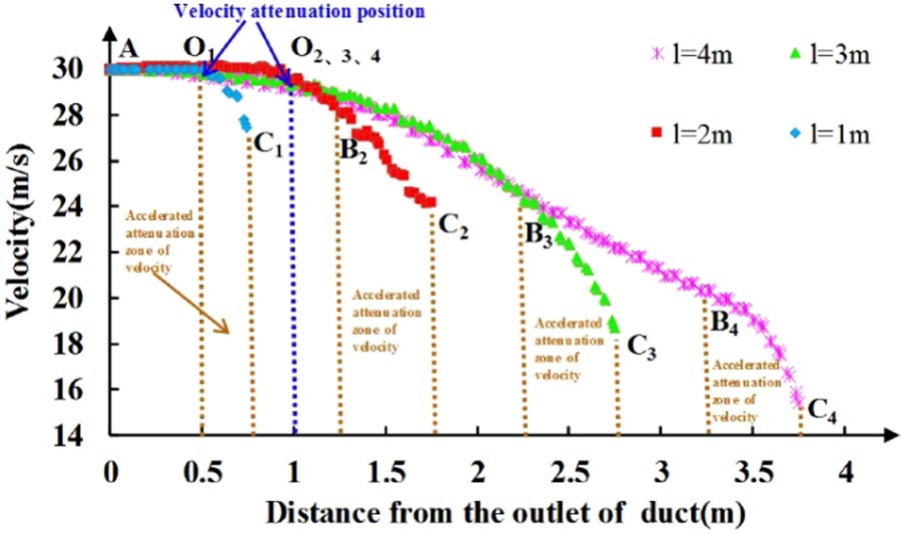
Curve of the axial velocity of ventilation duct in the jet zone at different positions of ventilation duct.

Figure 16 is a view of the wind flow trace of the section of the tunnel in the jet zone at different positions of the ventilation duct. From the view of the wind flow trace in the roadway, under the condition that the wind velocity of the ventilation duct and the height of the ventilation duct are constant, although the distance of the ventilation duct has little effect on the main body shape of the wind flow in the jet flow area, the influence on the wind velocity value of the roadway is relatively large. To this end, the axial velocity of the outlet of the ventilation duct to a distance of 0.25 m from the head is extracted under different ventilation duct position conditions, as shown in Fig. 17.

According to the wind velocity attenuation characteristic of the jet region in Fig. 17, the wind velocity in the jet region can be further divided into three regions: the original velocity region, the velocity attenuation region and the velocity decay acceleration region. The original velocity zone is the AO_1,2,3,4_ segments in the figure. In this region, the wind velocity value remains unchanged, and the wind velocity attenuation in the jet zone is not attenuated from the exit of the ventilation duct. This law is consistent with the theory of gas jets. When the distance of the ventilation duct is *l* ≥ 2 m, AO_2_ = AO_3_ = AO_4_ = 1 m, when the distance of the ventilation duct is *l* = 1 m, AO_1_ = 0.5 m, that is, when the distance of the ventilation duct is *l* ≥ 2 m, the range of the original velocity zone is independent of the distance of the ventilation duct, when the distance of the ventilation duct is *l* < 2 m, the range of the original velocity zone is related to the distance of the ventilation duct, which is mainly caused by the reverse airflow of the attachment area. The velocity decay zone is the segments O_2,3,4_B_2,3,4_ in the figure. In this region, the wind velocity value is attenuated by the resistance effect, and the range in the region increases with the increase of the distance of the ventilation duct; the velocity decay acceleration zone is the O_1_C_1_ and B_2,3,4_C_2,3,4_segments in the figure. In this region, the wind velocity decays faster due to the combined action of the reverse airflow and resistance in the attached area. It can be seen from the figure that B_2_C_2_ = B_3_C_3_ = B_4_C_4_ = 0.5 m and O_1_C_1_ = 0.25 m, that is, the influence of the reverse airflow in the adherent zone on the velocity acceleration zone is in the range of 0.25–0.5 m. According to the above analysis, it can be inferred that when the position of the ventilation duct is *l* ≤ 1.5 m, there is no velocity attenuation zone in the jet zone, that is, the original velocity zone directly transitions to the velocity attenuation acceleration zone; when the ventilation duct distance is *l* > 1.5 m, the jet zone is composed of an original velocity zone, a velocity decay zone, and a velocity decay acceleration zone. At the same time, it can be found in Fig. 17 that as the distance of the ventilation duct increases, the range of the jet region gradually increases, and the wind velocity at the end of the jet region gradually decreases, V_C1_ > V_C2_ > V_C3_ > V_C4_, that is, the airflow velocity entering the attachment area gradually decreases as the distance of the ventilation duct increases. Therefore, the position of the ventilation duct only affects the value of velocity in the attached jet zoon, but has little effect on its direction.

**Figure 18:**
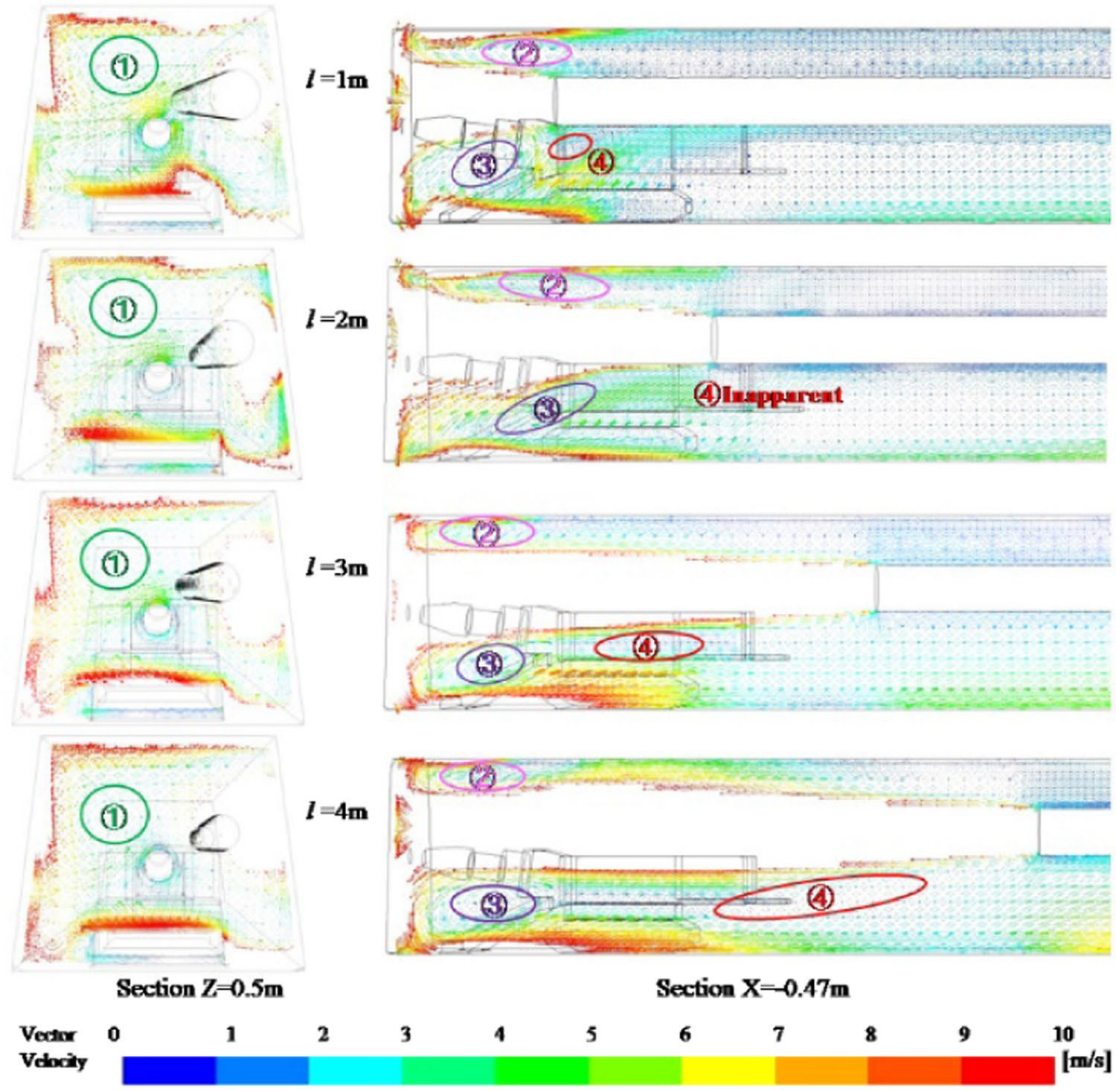
Vortex zone distribution variation law at different ventilation duct positions.

Since the formation of eddy current is caused by the entrainment action and space limitation of the high-velocity airflow, as the distance of the ventilation duct increases, the wind velocity in the roadway gradually decreases, which inevitably affects the vortex area in the roadway. Figure 18 is a vortex flow distribution map of the vortex area in the roadway of different ventilation ducts. In the figure, the position and range of the vortex flow of No. 1, 2, and 3 are basically unchanged, but the eddy current intensity gradually decreases as the wind velocity decreases. The NO.4 vortex flow near the ventilation duct mouth increases with the distance of the ventilation duct, and the eddy current range gradually increases, and the eddy current intensity gradually decreases. However, the eddy current intensity will gradually decrease as the velocity decays too fast. The vortex of No. 4 is not obvious when *l* = 2 m, which is mainly affected by the influence of No.3 vortex on the surrounding flow field structure, and the No. 4 eddy current is weakened. Therefore, the increase of the distance of the ventilation duct will lead to the expansion of the vortex area in the roadway, making the fluid in the front of the roadway more disorderly. At the same time, as the distance increases, the loss of fluid kinetic energy in the jet area gradually increases, which will also cause the wind velocity in the recirculation area behind the roadway to decrease.

**Figure 19:**
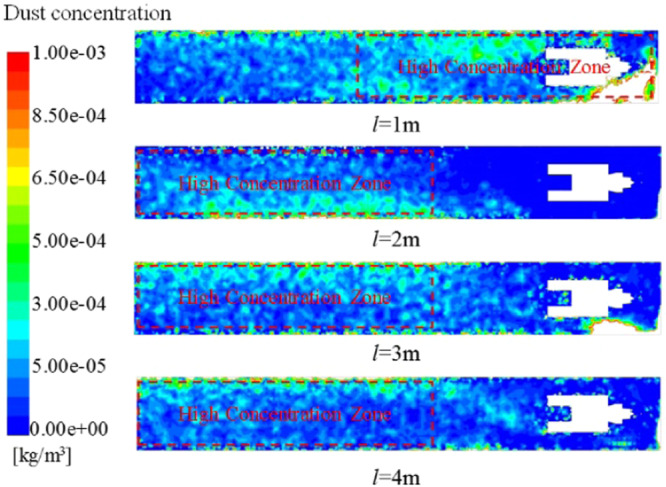
Distribution of dust concentration in roadway breathing zone at different outlet locations.

In order to study the influence of the position of the ventilation duct on the dust migration law in the roadway, the dust concentration distribution cloud map of the height of the breathing zone (Y = 0.45 m) was extracted (Fig. 19). It can be seen from the figure that no matter how far the ventilation duct is from the head position, the dust concentration in the rear breathing zone of the roadway is relatively high, but from the overall concentration distribution of the roadway. When the ventilation duct position *l* < 2 m, the dust concentration in the front half of the roadway relatively high, when the position of the ventilation duct *l* > 2 m, the dust concentration in the second half of the roadway is relatively high. When the position of the ventilation duct *l* = 2 m, the dust concentration in front of the roadway is the lowest, and the front of the roadway is the area where the underground workers are relatively concentrated, this is very beneficial for reducing dust damage to workers. Based on the influence of the position of the ventilation duct on the flow field partition in the roadway, the reason for this phenomenon can be analyzed: when the ventilation duct is close to the head, although the wind velocity is less attenuated in the jet region, the eddy current intensity formed in the vortex region is relatively large, so that most of the dust stays in the front half of the roadway and is not easily discharged; when the ventilation duct is far away from the working surface, the range of the vortex area in the roadway is increased, the dust is fully diffused under the action of the vortex, and the wind velocity in the roadway is relatively low, and the air carrying capacity of the dust is reduced, eventually the dust concentration in the second half of the roadway is relatively high. Therefore, considering the attenuation of wind velocity, structural characteristics of the flow field and dust removal efficiency, the position of the ventilation duct from the head should not be too close or too far. According to the above analysis, the distance of the ventilation duct is *l* = 2 m, the damage of dust to workers can be greatly reduced.

### Initial velocity of ventilation duct

When studying the influence of air velocity change on the flow field structure, dust migration and dust removal efficiency of the roadway, set the height of the ventilation duct *h* = 75 cm, and the distance between the ventilation duct and the working surface is *l* = 2 m. Therefore, the numerical simulation of the flow field structure and dust migration in the roadway under the conditions of air velocity *v* = 13 m/s, 20 m/s and 30 m/s is carried out.

**Figure 20:**
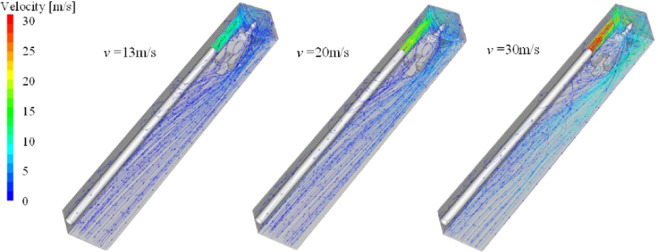
Wind flow trace of roadway under different inlet velocity.

**Figure 21:**
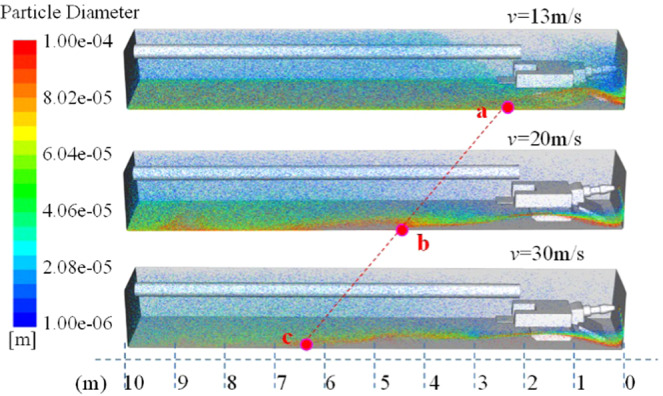
Distribution of dust particles at different wind velocity.

Figure 20 shows the wind flow trace diagram of the roadway under three different wind velocity. In the simulated wind velocity range, the flow field structure of the roadway does not change significantly, and the wind velocity in the roadway only differs in numerical value. Figure 21 is a distribution diagram of particles moving along the wind flow in the roadway. Points a, b, and c indicate the position of the particle group that first contacted the bottom plate with the wind flow movement, which are about 2.2 m, 4.3 m, and 6.2 m away from the head respectively. The higher the wind velocity, the farther the distance of the initial settlement of the dust group, and the dust intensity in the roadway space decreases significantly with the increase of the wind velocity in the roadway. It can be explained that the higher the wind velocity of the roadway, the stronger the ability of the wind flow to carry the particle group migration, the less the amount of dust that permeates the work space, the lower the damage to the workers. Therefore, in the range of wind velocity allowed by the roadway, the local ventilator with large air supply should be selected as much as possible to increase the dust-removing capacity of the working face and improve the air quality of the driving face.

## Conclusion

This paper focuses on the study of the flow field structure change law and dust migration law under the condition of different ventilation parameters. The main conclusions are as follows:

(1) The changing of the ventilation duct height affects the shape of the jet fluid, the wind velocity distribution in the attachment area, the distribution of the vortex structure, and the degree of disturbance of the wind flow in the recirculation zone. When the ventilation duct is located in the middle of the roadway, the wind flow is the most gentle, which is beneficial to the roadway dust discharge. Considering the distribution of wind velocity, flow field structure and dust distribution characteristics, when the height of the ventilation duct is *h* = 0.625 H (the height of the roadway), the ventilation and dust removal effect of the roadway is the best.

(2) In the range of the ventilation duct from the working surface *l* = 1~4 m, the farther the ventilation duct is from the working surface, the lower the wind velocity in the middle section of the roadway, which is unfavorable for ventilation and dust exhaust. The closer the ventilation duct is to the working surface, the greater the eddy current intensity in the roadway and the serious dust accumulation. When the ventilation duct is 2 m away from the head, the dust has the least damage to the workers in the roadway.

(3) The change of wind velocity in the roadway has a weak effect on the flow field, but an obvious improvement on dust removal. Within the scope of the study, the larger wind velocity shows a good ventilation and dedusting result. Therefore, the influence of the ventilation parameters of the roadway on the flow field and dust removal efficiency of the roadway should be considered in the design of roadway ventilation and dust removal.
